# Proton Versus Photon Radiotherapy for Non-Small Cell Lung Cancer: Updated Evidence from a Systematic Review and Meta-Analysis

**DOI:** 10.3390/cancers18030453

**Published:** 2026-01-30

**Authors:** Chiung-Chen Fang, Wen-Cheng Chen, Ming-Shao Tsai, Miao-Fen Chen

**Affiliations:** 1 Department of Radiation Oncology, Chang Gung Memorial Hospital, Chiayi, Chang Gung University, No. 6, Chia-Pu Rd., Chiayi 613, Taiwan; b9102060@cgmh.org.tw (C.-C.F.); danielchen@adm.cgmh.org.tw (W.-C.C.); 2 Department of Otorhinolaryngology, Head and Neck Surgery, Chang Gung Memorial Hospital, Chiayi, Chang Gung University, No. 6, Chia-Pu Rd., Chiayi 613, Taiwan; b87401061@cgmh.org.tw; 3 Department of Radiation Oncology, Chang Gung Memorial Hospital, Linkou, Chang Gung University, Taoyuan 333, Taiwan

**Keywords:** proton beam therapy, high-energy radiotherapy, non-small cell lung cancer, meta-analysis

## Abstract

Purpose: Proton beam therapy (PBT) offers superior dosimetric sparing of organs at risk compared to photon radiotherapy for non-small cell lung cancer (NSCLC); however, comparative clinical evidence regarding survival benefits remains conflicting. This systematic review and meta-analysis aimed to evaluate the clinical outcomes and toxicity profiles of PBT versus photon radiotherapy, with a specific focus on time-dependent survival patterns. Methods: We searched PubMed, EMBASE, and Cochrane CENTRAL databases for comparative studies published up to 10 October 2025. Primary outcomes were overall survival (OS), progression-free survival (PFS), and local progression-free survival (LPFS). Individual patient data (IPD) were reconstructed from Kaplan–Meier curves when hazard ratios (HRs) were not reported. Odds ratios (ORs) were calculated for survival at fixed time points (1, 3, and 5 years) and for toxicity endpoints. Results: Seven studies comprising 244,604 patients were included, encompassing retrospective cohorts, multi-institutional datasets, and one randomized trial. In the overall pooled analysis, PBT showed no statistically significant superiority over photon radiotherapy for OS (HR = 0.91, 95% CI: 0.69–1.19, *p* = 0.483), PFS (HR = 1.09, 95% CI: 0.81–1.47, *p* = 0.572), or LPFS (HR = 0.89, 95% CI: 0.47–1.69, *p* = 0.732). Sensitivity and subgroup analyses restricted to Stage I and Stage I–II NSCLC similarly failed to demonstrate significant differences in survival outcomes. However, exploratory time point analysis utilizing ORs revealed a distinct temporal pattern: PBT was associated with improved odds of all-cause mortality at 1 year (OR = 0.60, 95% CI: 0.49–0.73, *p* < 0.001). This survival advantage dissipated over time, with no significant differences observed at 3 years or 5 years. Regarding safety, PBT did not significantly reduce the odds of grade ≥ 2 radiation pneumonitis (OR = 0.98, 95% CI: 0.41–2.33, *p* = 0.967) or grade ≥ 3 events (OR = 1.40, *p* = 0.540) compared to photons. Conclusions: While long-term oncologic control appears comparable between proton and photon radiotherapy, exploratory analyses suggest that PBT is associated with improved odds of 1-year overall survival. This potential early benefit, observed in retrospective cohorts, likely reflects the mitigation of acute treatment-related mortality. These findings are hypothesis-generating and support the use of PBT for patients at high risk of toxicity and advocate for a model-based approach to patient selection.

## Simple Summary

Non-small cell lung cancer (NSCLC) is a major cause of cancer-related mortality. While radiotherapy is a standard treatment, traditional photon (X-ray) radiation can inadvertently expose healthy organs, such as the heart and lungs, to excess dose. Proton beam therapy (PBT) allows for more precise radiation delivery, potentially reducing the risk of side effects. In this systematic review and meta-analysis of seven studies involving over 240,000 patients, we compared the clinical outcomes of PBT versus photon radiotherapy. Our analysis indicated that while long-term survival rates appeared similar between the two modalities, PBT was associated with improved odds of survival during the first year following treatment. These findings are hypothesis-generating and suggest that PBT might be a valuable option for patients who are frail or at high risk of toxicity, potentially helping to mitigate early treatment-related mortality.

## 1. Introduction

Lung cancer is the second most common cancer for both men and women worldwide [1]. Among different types of lung cancer, non-small cell lung cancer (NSCLC) is the most common epithelial lung cancer and accounts for approximately 85% of all lung cancer cases [2]. The estimated 5-year survival of NSCLC is approximately 26.4% for all stages, remaining a leading cause of cancer-related mortality worldwide [3]. Despite advances in screening and systemic therapies, about 30% of NSCLC cases are diagnosed at a locally advanced stage [4]. This necessitates treatments that are both curative and capable of extending life span. Concurrent chemoradiotherapy is a cornerstone of management for stage III disease, and radiotherapy remains essential for patients who are medically inoperable or have residual disease after systemic treatment [5,6].

Radiotherapy for NSCLC has traditionally relied on photon-based techniques, including three-dimensional conformal radiotherapy (3D-CRT), intensity-modulated radiotherapy (IMRT), and volumetric modulated arc therapy (VMAT) [7,8]. These modalities deliver therapeutic radiation dosages but inevitably expose surrounding healthy tissues to excess radiation, resulting in an unavoidable “exit dose” and a “low-dose bath” spread across the thoracic cavity. This integral dose inadvertently irradiates critical structures such as the lungs, heart, esophagus, and bone marrow, potentially leading to severe toxicities that compromise patients’ physiological reserve and ability to complete systemic therapies [9,10].

Proton beam therapy (PBT), by contrast, uses charged particles with unique depth-dose characteristics (the Bragg peak) that allow for reduced exit dose and improved normal-tissue sparing [11,12]. Dosimetric studies have consistently shown that PBT reduces mean doses to the heart and lungs compared to IMRT, potentially widening the therapeutic window [13]. PBT has been used in NSCLC treatment and showed acceptable rates of tumor control and survival [14,15]. As technological advances have improved proton delivery and availability, the number of patients receiving PBT is increasing [16].

However, translating this physical superiority into proven clinical survival benefits has been challenging. Randomized controlled trials and retrospective analyses have yielded conflicting results regarding survival advantages and toxicity reduction [17,18]. Given the expanding use of PBT and an increasing body of comparative evidence, an updated synthesis of outcome data is needed. This systematic review and meta-analysis aimed to evaluate the most recent evidence comparing proton versus photon radiotherapy for patients with NSCLC, focusing on survival outcomes at specific time points (1, 3, and 5 years), treatment-related toxicity, and dosimetric advantages.

## 2. Methods

This present systematic review and meta-analysis was conducted in accordance with the Preferred Reporting Items for Systematic Reviews and Meta-Analyses (PRISMA) guidelines [19]. A literature search was conducted through major public databases (i.e., PubMed, EMBASE, and Cochrane CENTRAL) using the keywords “non-small cell lung cancer”, “proton therapy”, “photon therapy”, and “survival disease”, among others, combined with Boolean operators and using Medical Subject Headings (MeSH) terms where appropriate for studies published from inception up to 10 October 2025. No restriction on clinical stage was applied at the search level; stage-specific subgroup analyses were performed to address clinical heterogeneity. Specific terms such as “stage I” and “early-stage” were included to maximize sensitivity for smaller cohorts that might not be indexed under broad “NSCLC” terms, though the inclusion criteria encompassed all clinical stages. The search string used for the databases was:

(“Carcinoma, Non-Small-Cell Lung”[Mesh] OR “non-small cell lung cancer”[Title/Abstract] OR NSCLC[Title/Abstract])

AND

(“stage I”[Title/Abstract] OR “stage II”[Title/Abstract] OR “early-stage”[Title/Abstract])

AND

(“Proton Therapy”[Mesh] OR “proton therapy”[Title/Abstract])

AND

(“Radiotherapy”[Mesh] OR “photon therapy”[Title/Abstract] OR “photon radiotherapy”[Title/Abstract] OR “3D-CRT”[Title/Abstract] OR “IMRT”[Title/Abstract] OR “stereotactic body radiotherapy”[Title/Abstract] OR SBRT[Title/Abstract])

AND

(“Survival”[Mesh] OR “Disease-Free Survival”[Mesh] OR “Quality of Life”[Mesh] OR “overall survival”[Title/Abstract] OR OS[Title/Abstract] OR “progression-free survival”[Title/Abstract] OR PFS[Title/Abstract] OR “local control”[Title/Abstract] OR toxicity [Title/Abstract])

In addition, the reference lists of included studies were hand-searched to identify other potentially relevant studies.

### 2.1. Selection Criteria

This systematic review and meta-analysis was performed in accordance with the PICOS criteria (participants, intervention, comparison, outcomes, study design). The study population consisted of adult patients with NSCLC who underwent radiotherapy as definitive treatment, either due to medical inoperability or as an alternative to surgical resection. The intervention of interest was PBT, including both conventionally fractionated PBT and proton-based stereotactic body radiotherapy (SBRT). The comparator group included photon-based radiotherapy modalities, such as 3D-CRT, IMRT, and photon-based SBRT. Eligible studies were required to report at least one of the following outcomes: overall survival (OS), disease-free survival (DFS) or progression-free survival (PFS), local control rate, radiation-induced toxicities (e.g., pulmonary, esophageal, or cardiac toxicities graded according to CTCAE or equivalent criteria), or quality of life (QoL). Only randomized controlled trials (RCTs) and two-arm comparative prospective or retrospective studies were included.

Studies were excluded if they were non-comparative or single-arm studies, reviews, meta-analyses, editorials, letters, comments, conference abstracts, case reports, proceedings, or personal communications. Studies that did not report quantitative outcomes of interest were also excluded. Additionally, studies comparing different photon-based radiotherapy techniques with each other or different PBT techniques with each other, as well as non-human studies, were not considered eligible.

This systematic review and meta-analysis is registered in the International Prospective Register of Systematic Reviews (PROSPERO) under the registration number CRD420261279661.

### 2.2. Main Outcome Measures and Data Extraction

The primary outcomes of interest were time-to-event endpoints assessed using hazard ratios (HRs) with corresponding 95% confidence intervals (CIs). These outcomes included OS, defined as the time from initiation of radiotherapy to death from any cause; PFS, defined as the time from treatment initiation to disease progression or distant metastasis; and local progression-free survival (LPFS), defined as the time from treatment initiation to local tumor recurrence or progression within the irradiated field.

In addition to HR-based analyses, 1-year point survival rates for OS, PFS, and LPFS were extracted or calculated when available and were analyzed as secondary time-specific outcomes to facilitate comparison across studies with heterogeneous follow-up durations. When HRs or 1-year survival rates were not directly reported, they were estimated from Kaplan–Meier curves using established methods. Individual patient data (IPD) reconstruction was performed to enable the estimation of HRs where they were not explicitly reported and to allow for time-specific survival probability estimations (e.g., 1-year survival) that were not provided in tabular form.

Safety outcomes included treatment-related adverse events (TRAEs) and radiation pneumonitis (RP), which were analyzed according to severity grade. The incidence of grade ≥ 2 and grade ≥ 3 TRAEs and RP was extracted based on the Common Terminology Criteria for Adverse Events (CTCAE) or equivalent grading systems. When safety outcomes were not directly reported in tabular form, relevant data were derived from the text or Supplementary Materials of the included studies where feasible.

Data were independently extracted from each eligible study using a standardized data collection form. The following study characteristics were recorded: first author and year of publication; country or region; study design (randomized controlled trial, prospective cohort, retrospective cohort, or case–control study); data source (hospital-based records, institutional registries, or administrative databases); and study period. Patient baseline characteristics extracted included age, sex distribution, clinical stage (e.g., stage 0, I, IIA), tumor location (central versus peripheral), and other reported baseline factors such as comorbidities, pulmonary function parameters, and Eastern Cooperative Oncology Group (ECOG) performance status. Treatment-related variables included radiotherapy modality (PBT (e.g., pencil beam scanning, passive scattering) or photon-based techniques (e.g., three-dimensional conformal radiotherapy, intensity-modulated radiotherapy, stereotactic body radiotherapy)), radiation dose and fractionation schedules (total dose, dose per fraction, and number of fractions), use of concurrent or adjuvant therapies (chemotherapy, immunotherapy, or other systemic treatments), and duration of follow-up. Outcome data relevant to efficacy and safety analyses were also extracted when available. Any discrepancies in data extraction were resolved through discussion or consultation with a third reviewer.

### 2.3. Ethics Statement

This systematic review and meta-analysis of published studies neither required nor used raw patient data and private information, therefore approval of the protocol by the hospital institutional review board (IRB) and informed consent from study subjects were waived.

### 2.4. Risk of Bias Assessment

The quality of included studies was assessed using the Cochrane Collaboration tool [20]. While this tool is primarily designed for randomized trials, it was adapted to assess bias domains in the included observational studies, with the acknowledgement that retrospective designs inherently carry a higher risk of selection and performance bias. This tool assesses risk of bias via the following seven criteria: selection bias (random sequence generation and allocation concealment), performance bias (blinding of participants and personnel), detection bias (blinding of outcome assessment), attrition bias (incomplete outcome), reporting bias (selective outcome reporting), and other bias (inclusion of intention-to-treat analysis). Quality assessment was performed by two independent reviewers, and a third reviewer was consulted if any uncertainties occurred.

### 2.5. Statistical Analysis

In this meta-analysis, the primary outcomes were OS, PFS, and LPFS. Secondary outcomes included radiation pneumonitis and musculoskeletal toxicity. Primary outcomes were pooled using inverse-variance-weighted hazard ratios under fixed- or random-effects models, with HRs and corresponding 95% CIs as the effect sizes.

For studies which presented Kaplan–Meier (K-M) curves without reporting HRs [21,22,23], the R package IPDfromKM (version 0.1.10) within the R statistical software environment (version 4.5.1) [24] was used to extract raw coordinate data from published K-M curves and reconstruct individual patient data (IPD) from the extracted coordinates. HRs were further estimated from the reconstructed IPD. The accuracy of reconstruction was evaluated using predefined thresholds, including root mean square error (RMSE) ≤ 0.05, mean absolute error ≤ 0.02, max absolute error ≤ 0.05, and a large *p*-value of the Kolmogorov–Smirnov test.

As the included studies did not report event counts at fixed time points (1, 3, and 5 years), cumulative event rates were approximated as the complement of the survival probability [1 − S(t)] at each time point. Estimated event counts were obtained by multiplying the event rate by the number of patients in each treatment arm, which were further used to calculate odds ratios (ORs). Secondary outcomes, including radiation pneumonitis and musculoskeletal toxicity, were summarized as ORs based on reported event rates. For studies with single-arm zero events, the Haldane–Anscombe continuity correction was applied by adding 0.5 to each cell of the 2 × 2 table prior to OR calculation [21,25].

Heterogeneity among the studies was evaluated using the Cochran Q test and I^2^ statistic, categorized as low (I^2^ < 25%), moderate (25% ≤ I^2^ < 50%), substantial (50% ≤ I^2^ < 75%), and high (I^2^ ≥ 75%) [26,27]. A random-effects model was applied when I^2^ exceeded 50% or when fewer than four studies contributed to an outcome; otherwise, a fixed-effects model was used. Sensitivity analyses were performed using a leave-one-out approach to evaluate the robustness of pooled estimates. A two-sided *p*-value of <0.05 was regarded as statistically significant. All analyses were conducted using Comprehensive Meta-Analysis software (CMA, version 3).

## 3. Results

### 3.1. Study Selection

**Figure 1:**
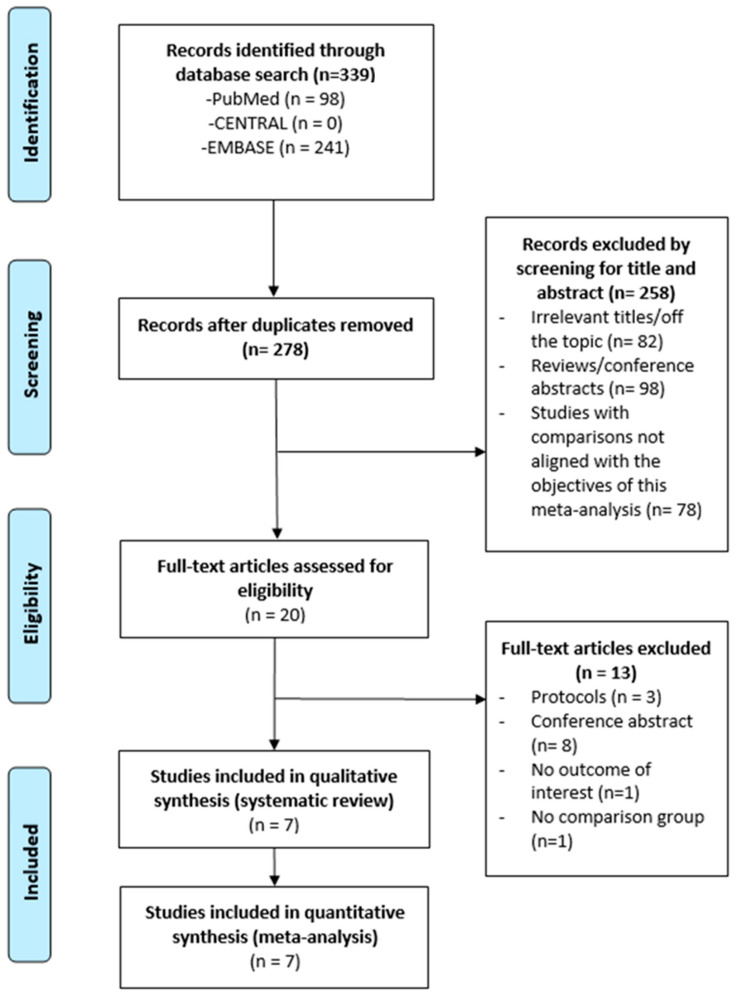
Flow chart for selection of publication.

A total of 20 full-text articles were assessed for eligibility, and 13 were excluded. Ultimately, 7 studies were included in this meta-analysis (Figure 1), comprising a total of 244,604 patients.

### 3.2. Characteristics of Included Studies

Table 1 summarizes the characteristics of the seven included studies published between 2017 and 2022. These studies included retrospective single-institution cohorts, multi-institutional datasets, one randomized clinical trial, and one large database analysis. Study periods ranged from 2004 to 2019, with sample sizes varying widely across studies from 19 to 243,822 patients. Photon radiotherapy consisted of various modalities, including SBRT, 3D-CRT, and IMRT, whereas proton beam therapy (PBT) techniques included passive scattering, intensity-modulated proton therapy (IMPT), and stereotactic body proton therapy (SBPT). The prescribed radiation dose was typically 60 Gy, delivered in 4–30 fractions depending on technique and institutional practice. Median or mean patient age ranged from 67 to 76 years, and the proportion of male patients varied from 52.1% to 96.7%.

Across the included studies, most patients had stage I–II non-small cell lung cancer (NSCLC). Tumor locations varied across cohorts, including peripheral, central, and chest wall-adjacent lesions. Baseline characteristics were heterogeneous: 70–85% of patients had an ECOG performance status of 0–1, while comorbidity profiles—including COPD, ILD, CAD, and elevated Charlson comorbidity scores—differed substantially. Adenocarcinoma and squamous cell carcinoma were the predominant histologic subtypes. Two studies reported concurrent chemotherapy use in 58–87% of patients, whereas other studies did not report systemic therapy. Median follow-up duration ranged from approximately 10 to 60 months.

Table 2 summarizes radiation pneumonitis and other treatment-related toxicities. Rates of grade ≥ 2 radiation pneumonitis were 10.8–19.6% in photon cohorts and 4.3–26.4% in proton cohorts, whereas grade ≥ 3 events were generally uncommon, occurring in 1.1–11.9% and 0–17.6% of patients, respectively. Rib fracture and chest wall pain were more frequently reported in photon-treated patients (16.2–24.7%) compared with proton-treated patients (4.3–15.1%). Skin and esophageal toxicities were inconsistently reported and were overall infrequent.

### 3.3. Meta-Analysis

**Figure 2:**
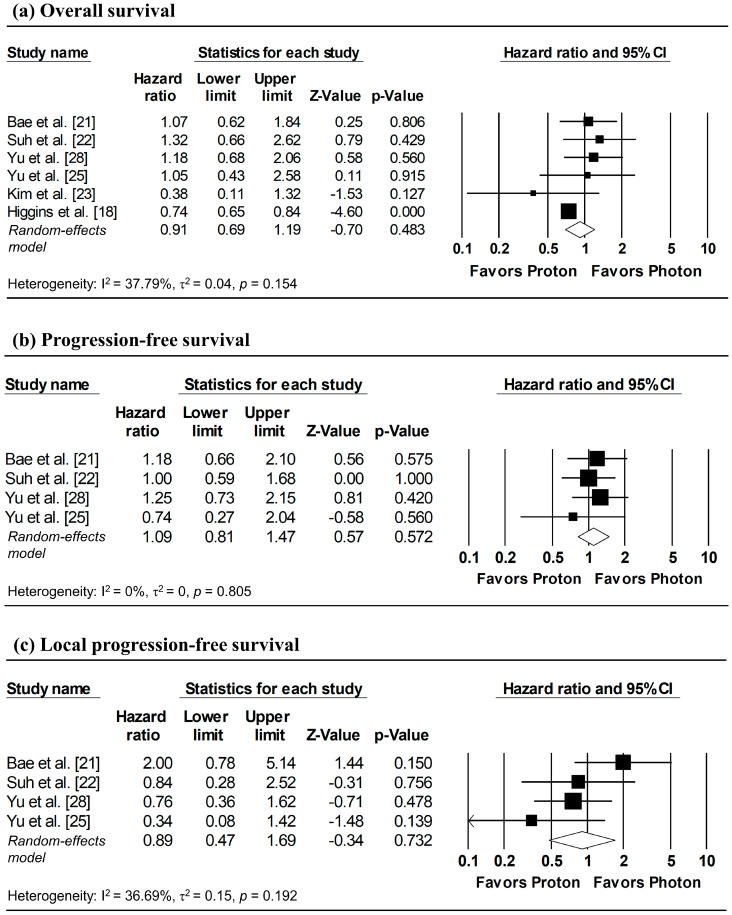
Forest plots comparing proton versus photon radiotherapy for (**a**) overall survival, (**b**) progression-free survival, and (**c**) local progression-free survival. Note: Analyses for Bae et al. [21], Suh et al. [22], and Kim et al. [23] utilized IPD reconstruction, while other studies used directly reported data. Z-values reported as “0.00” correspond to values < 0.01, whereas *p*-values reported as “0.000” correspond to values < 0.001.

The results of the meta-analysis and corresponding forest plots for OS, PFS, and LPFS comparing proton versus photon radiotherapy are presented in Figure 2. HRs for three studies—Bae et al. [21], Suh et al. [22], and Kim et al. [23]—were estimated from reconstructed IPD, with the analyses of Suh et al. [22] based on matched cohorts.

#### 3.3.1. Overall Survival

Three studies reported HRs for OS, while HRs for the remaining studies were estimated from reconstructed IPD [21,22,23]. Although between-study heterogeneity was modest (I^2^ = 37.79%), a random-effects model was applied due to methodological differences of the database-based study by Higgins et al. [18] relative to other studies. The pooled analysis showed no significant difference in overall mortality between proton and photon radiotherapy (pooled HR = 0.91, 95% CI: 0.69–1.19, *p* = 0.483; Figure 2a).

#### 3.3.2. Progression-Free Survival

Two studies directly reported HRs for PFS, while HRs for the remaining studies were estimated from reconstructed IPD [21,22]. Despite the absence of heterogeneity (I^2^ = 0%), a random-effects model was used due to the limited number of included studies (≤ 4). The pooled analysis revealed no significant difference in disease progression between proton and photon radiotherapy (pooled HR = 1.09, 95% CI: 0.81–1.47, *p* = 0.572; Figure 2b).

#### 3.3.3. Local Progression-Free Survival

HRs for LPFS were available from two studies, with additional HRs derived from reconstructed IPD [21,22]. Despite low heterogeneity across studies (I^2^ = 36.69%), a random-effects model was applied due to the small number of included studies. No significant difference in local tumor control was observed between proton and photon radiotherapy (pooled HR = 0.89, 95% CI; 0.47–1.69, *p* = 0.732; Figure 2c).

### 3.4. Sensitivity Analyses

Table 3 presents leave-one-out sensitivity analyses for OS, PFS, and LPFS. Sequential exclusion of each study did not alter the pooled HRs, which remained non-significant across all outcomes. The results indicated that this meta-analysis had good reliability and was not overly influenced by any single study.

### 3.5. Subgroup Analyses

**Figure 3:**
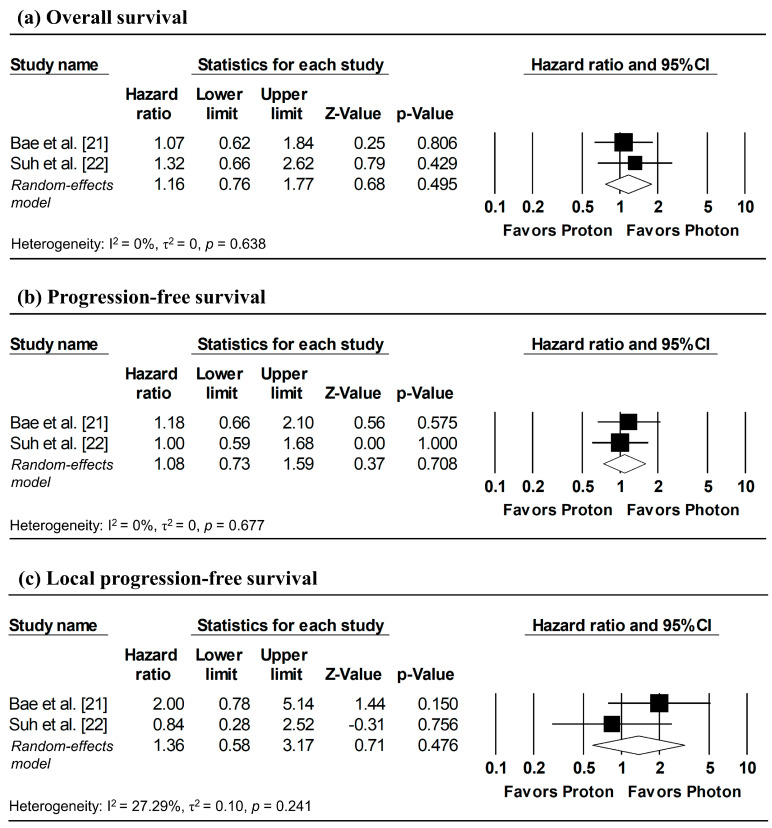
Forest plots comparing proton versus photon radiotherapy for (**a**) overall survival, (**b**) progression-free survival, and (**c**) local progression-free survival in Stage I NSCLC studies. Z-values reported as “0.00” correspond to values < 0.01.

**Figure 4:**
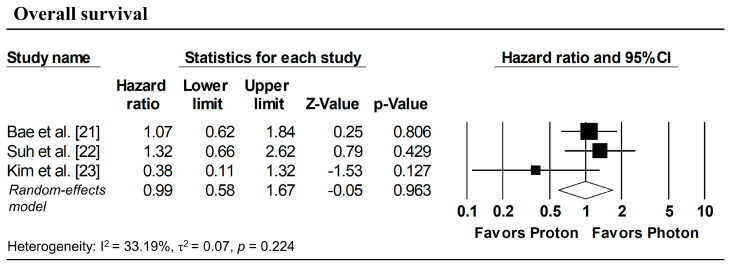
Forest plots comparing proton versus photon radiotherapy for overall survival in Stage I–II NSCLC studies.

Figure 3 presents the pooled analyses restricted to studies enrolling patients with stage I NSCLC. No significant differences were observed between proton and photon radiotherapy in OS (pooled HR = 1.16, 95% CI: 0.76–1.77, *p* = 0.495; Figure 3a), PFS (pooled HR = 1.08, 95% CI: 0.73–1.59, *p* = 0.708; Figure 3b), and LPFS (pooled HR = 1.36, 95% CI: 0.58–3.17, *p* = 0.476; Figure 3c). Similarly, in Stage I–II NSCLC studies, no significant difference in OS was observed (pooled HR = 0.99, 95% CI: 0.58–1.67, *p* = 0.963; Figure 4).

### 3.6. Incidence of Overall Mortality, Progression, and Local Progression at 1, 3, and 5-Year Follow-Up

**Figure 5:**
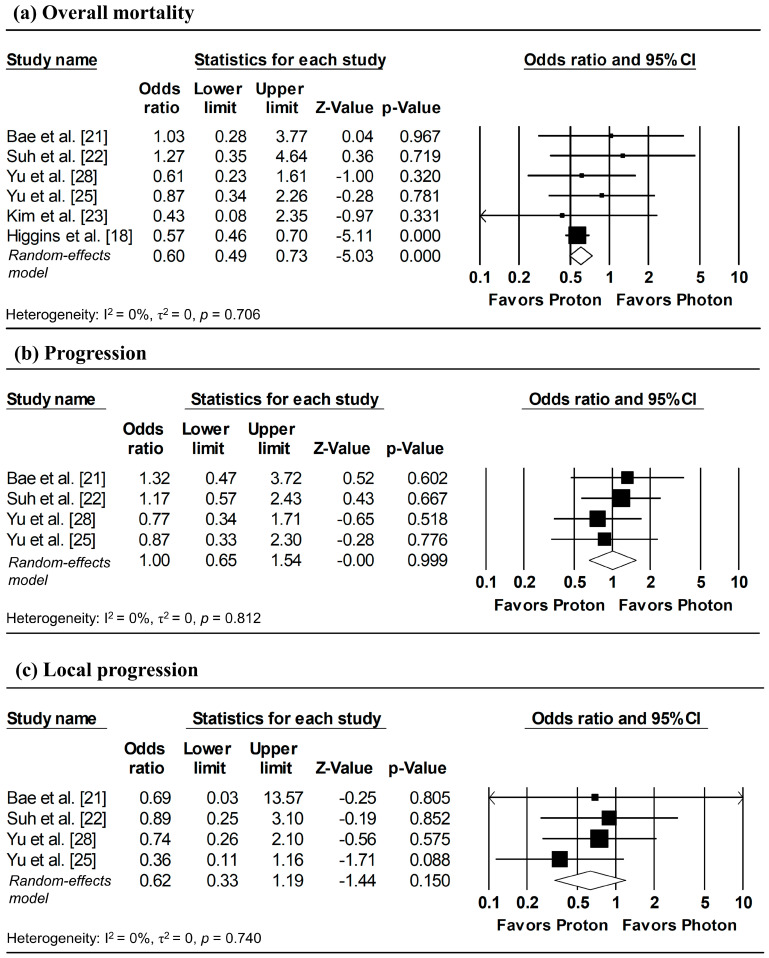
Forest plots of odds ratios comparing proton versus photon radiotherapy for (**a**) overall mortality, (**b**) progression, and (**c**) local progression at 1-year, derived from Kaplan–Meier estimates. Z-values reported as “0.00” correspond to values < 0.01, whereas *p*-values reported as “0.000” correspond to values < 0.001.

**Figure 6:**
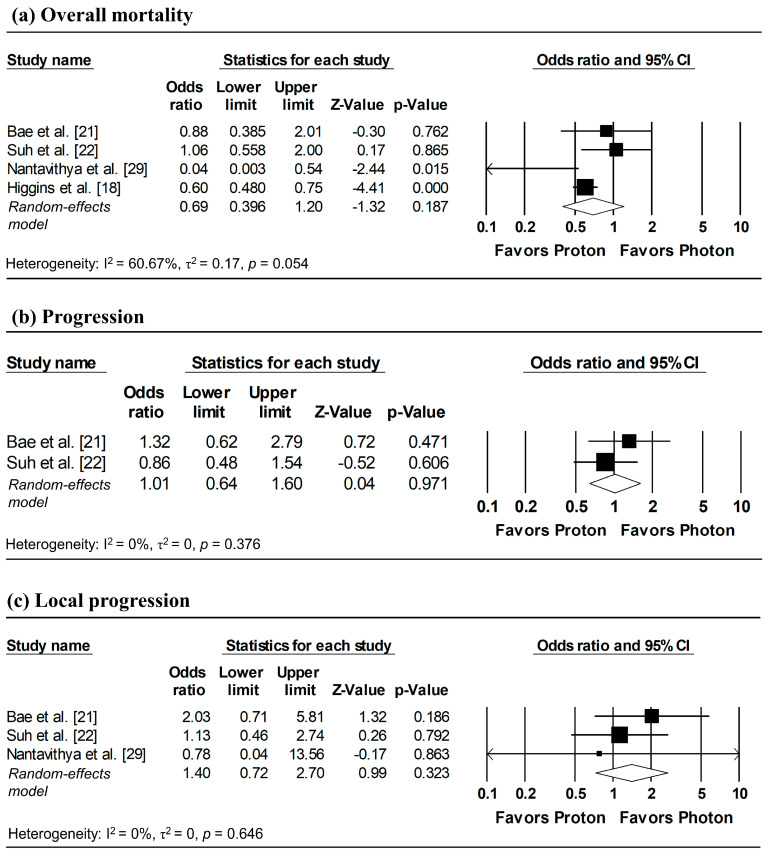
Forest plots of odds ratios comparing proton versus photon radiotherapy for (**a**) overall mortality, (**b**) progression, and (**c**) local progression at 3-year, derived from Kaplan–Meier estimates. *p*-values reported as “0.000” correspond to values < 0.001.

**Figure 7:**
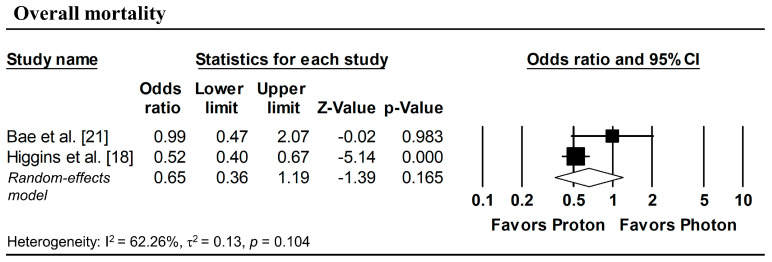
Forest plots of odds ratios comparing proton versus photon radiotherapy for overall mortality at 5-year, derived from Kaplan–Meier estimates. *p*-values reported as “0.000” correspond to values < 0.001.

To estimate ORs at fixed time points (1, 3, and 5 years), survival probabilities derived from K-M curves were used. All survival probabilities for Bae et al. [21] and Suh et al. [22], as well as the 3-year OS probability for Higgins et al. [18], were extracted from the published K-M curves, whereas the remaining survival probabilities were obtained directly from the original publications. The pooled analysis showed a significantly lower odds of overall mortality at 1 year with proton radiotherapy compared with photon radiotherapy (pooled OR = 0.60, 95% CI: 0.49–0.73, *p* < 0.001; Figure 5a), with no significant differences observed in 1-year disease progression and local progression (Figure 5b,c). At longer follow-up intervals, there were no significant differences in the 3-year odds of overall mortality, progression, and local progression, nor in 5-year overall mortality (Figure 6 and Figure 7).

### 3.7. Adverse Events

**Figure 8:**
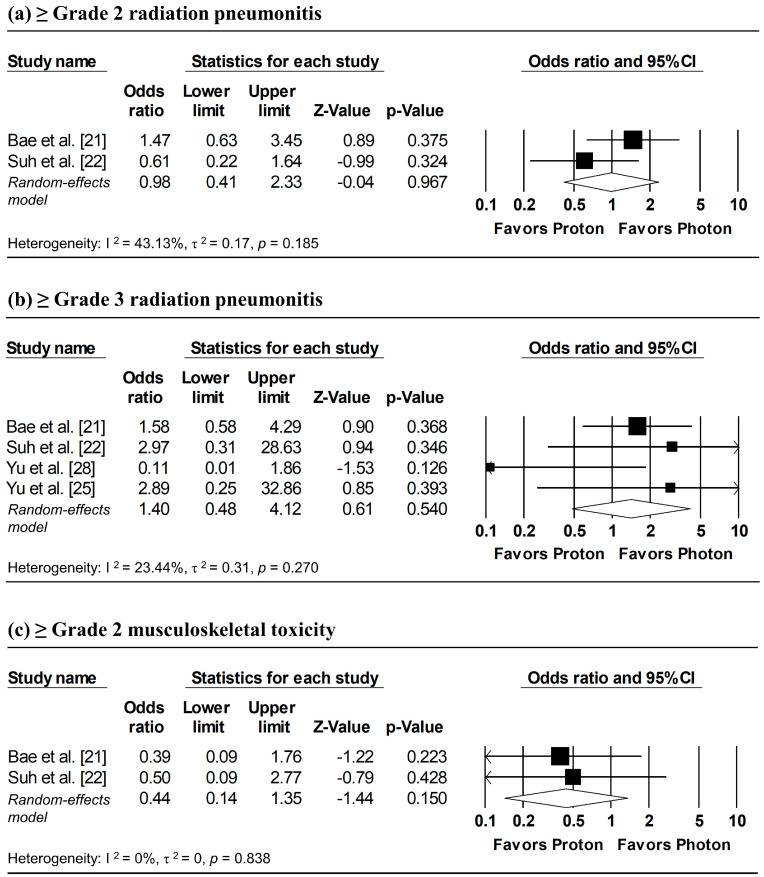
Forest plots of odds ratios comparing proton versus photon radiotherapy for (**a**) ≥ grade 2 radiation pneumonitis, (**b**) ≥ grade 3 radiation pneumonitis, and (**c**) ≥ grade 2 musculoskeletal toxicity.

Figure 8 presents the pooled analyses of radiation pneumonitis and musculoskeletal toxicity. Random-effects meta-analyses showed no significant differences between proton and photon radiotherapy in the incidence of ≥grade 2 radiation pneumonitis (pooled OR = 0.98, 95% CI: 0.41–2.33, *p* = 0.967), ≥grade 3 radiation pneumonitis (pooled OR = 1.40, 95% CI: 0.48–4.12, *p* = 0.540), and ≥grade 2 musculoskeletal toxicity (pooled OR = 0.44, 95% CI: 0.14–1.35, *p* = 0.150). Overall, the findings suggest comparable safety between proton and photon radiotherapy.

## 4. Discussion

### 4.1. Overview and Contextualization

Radiotherapy plays a cornerstone role in the curative management of NSCLC. Historically, the introduction of advanced photon-based techniques, such as IMRT, has improved target conformity; however, the “low-dose bath” delivered to surrounding healthy tissues remains an inherent physical limitation. The clinical rationale for PBT has long been predicated on the Bragg peak effect, theoretically enabling superior sparing of critical intrathoracic organs. Despite these undisputed dosimetric advantages, translating physical superiority into tangible clinical survival benefits has proven challenging in previous trials [17]. Against this backdrop, our systematic review and meta-analysis sought to elucidate whether the modern application of PBT translates into a measurable survival advantage. By synthesizing data from over 240,000 patients, our findings provide a nuanced answer: while long-term survival remains comparable to photon therapy, PBT was associated with improved odds of survival in the first year following treatment in our exploratory time point analysis. It is important to note that this finding is derived from ORs at fixed time points rather than hazard ratios from time-to-event modeling, and thus should be interpreted as hypothesis-generating.

### 4.2. The “Acute Window”: Interpreting the 1-Year Survival Benefit

The observation that PBT significantly improves 1-year overall survival (OR = 0.60, *p* < 0.001) without altering long-term outcomes presents a distinct temporal pattern. This finding aligns with the hypothesis that PBT acts primarily by mitigating treatment-related mortality (TRM) during the acute and subacute phases. In locally advanced NSCLC, early mortality is frequently driven by severe radiation-induced toxicities—such as pneumonitis, cardiac decompensation, or complications arising from esophagitis—rather than immediate tumor progression [30].

Our results suggest that PBT effectively guides patients through this high-risk “acute toxicity window.” By reducing the integral dose to the functional lung, heart, and esophagus, PBT preserves the patient’s physiological reserve. However, as patients survive beyond this initial phase, the primary driver of mortality shifts to the natural biology of the disease (i.e., distant metastasis). This convergence of survival curves at 3 and 5 years is consistent with the findings from the randomized trial by Liao et al. [17]. Furthermore, as patients survive beyond the acute phase, the primary driver of mortality likely shifts to distant metastasis, where local modality has limited impact. This interpretation is supported by long-term data from the Phase III PACIFIC trial, which established that distant metastasis remains the predominant pattern of failure in locally advanced NSCLC [31]. The 5-year outcomes demonstrated that the addition of systemic immunotherapy (durvalumab) significantly improved survival by reducing new distant lesions, highlighting that long-term prognostication is increasingly driven by systemic disease control [31,32]. Our data imply that PBT buys time and safety upfront, which is a critical endpoint in itself for patients with significant comorbidities.

### 4.3. Esophageal Toxicity and the Nutritional Cascade

A critical mechanism likely underpinning the 1-year survival advantage is the reduction in severe esophagitis. Although our pooled analysis of toxicity was limited by reporting heterogeneity, the clinical consensus, supported by dosimetric studies such as those by Cushman et al., indicates that PBT significantly reduces the mean esophageal dose compared to IMRT [33]. In standard photon chemoradiotherapy, grade 3 acute esophagitis is a debilitating complication that often necessitates feeding tube placement and treatment interruptions.

The “nutritional cascade” hypothesis posits that by sparing the esophagus, PBT allows patients to maintain oral intake and prevent severe weight loss and sarcopenia. Sarcopenia has been identified in multiple studies as an independent poor prognostic factor in NSCLC [34]. Furthermore, maintaining a robust performance status is a prerequisite for completing concurrent chemotherapy and, more importantly, for initiating and sustaining consolidation immunotherapy (e.g., Durvalumab), which is now the standard of care following the PACIFIC trial [35]. By minimizing “collateral damage” to the aerodigestive tract, PBT may indirectly enhance the efficacy of multimodal treatment.

### 4.4. The Cardiac Component: Echoes of RTOG 0617

The potential contribution of cardiac sparing to the observed early survival benefit cannot be overstated. The landmark RTOG 0617 trial inadvertently revealed that higher radiation doses to the heart were associated with worse overall survival, independent of tumor control [9]. Photon-based IMRT/VMAT, while sparing the spinal cord, often deposits a low-dose bath across the heart. In contrast, PBT eliminates the exit dose, significantly reducing the mean heart dose and V30 parameters. It is biologically plausible that the reduction in subclinical cardiac injury—such as microvascular damage, pericardial effusion, or conduction abnormalities—contributes to lower non-cancer mortality in the first 12 months [36].

### 4.5. The Pneumonitis Paradox: Physics vs. Biology

Interestingly, our meta-analysis found no statistically significant difference in the rates of grade ≥ 2 radiation pneumonitis between proton and photon cohorts. This finding mirrors the results of the MD Anderson randomized trial (Liao et al.), which also failed to demonstrate a reduction in clinical pneumonitis despite superior lung dosimetry [17]. This “pneumonitis paradox” may be explained by two factors. First, the rapid evolution of photon techniques, such as VMAT, has significantly improved lung sparing compared to older 3D-CRT techniques, narrowing the therapeutic ratio [8]. Second, and perhaps more importantly, is the uncertainty regarding the Relative Biological Effectiveness (RBE) of protons. While clinical practice assumes a constant RBE of 1.1, radiobiological data suggest that the RBE increases at the distal end of the Bragg peak due to high Linear Energy Transfer (LET) [37]. If the distal edge of the proton beam terminates within the lung parenchyma, the localized biological damage may be greater than predicted by physical dose alone, potentially offsetting the benefit of reduced V5 and V20.

While our pooled analysis did not reveal statistically significant differences in high-grade toxicities between PBT and photon therapy, these findings must be interpreted with caution. The lack of observed difference should not be conflated with equivalence. Toxicity reporting varied widely across included studies, with inconsistent grading criteria and follow-up durations. Consequently, subtle but clinically relevant differences, particularly regarding low-grade or organ-specific toxicities (e.g., cardiac events), may be underrepresented in the current literature.

### 4.6. Immunomodulation: The Lymphocyte-Sparing Hypothesis

Finally, an emerging explanation for the survival advantage is the preservation of the host immune system. Circulating lymphocytes are exquisitely radiosensitive, and severe radiation-induced lymphopenia (RIL) has been correlated with inferior survival in NSCLC, particularly in the era of immunotherapy [38,39]. Because photons deliver a low-dose bath to a large volume of the body, they are more likely to deplete lymphocytes than PBT. The “lymphocyte-sparing” effect of protons may preserve the patient’s immune competence, creating a synergistic environment for immune checkpoint inhibitors to function.

### 4.7. The Prognostic Dominance of Physiological Reserve and Systemic Control

While our analysis highlights the dosimetric and early survival advantages of PBT, the lack of significant difference in long-term survival (3- and 5-year OS) must be interpreted within the broader physiological and oncological context of NSCLC. Unlike other malignancies, lung cancer compromises the respiratory system, a vital organ whose function is directly linked to immediate survival. Consequently, the patient’s initial Performance Status (PS) serves as the single most critical determinant of short-term mortality, often outweighing specific treatment modalities [40,41]. The 1-year survival benefit observed in our PBT group likely reflects the preservation of this fragile physiological reserve.

However, NSCLC remains an inherently systemic disease. The landscape of lung cancer management has been revolutionized by advancements in systemic therapies, including tyrosine kinase inhibitors (TKIs) and immune checkpoint inhibitors (ICIs) [42,43]. In this modern paradigm, the efficacy of targeted therapies and immunotherapy often dictates the long-term ceiling of survival, potentially diluting the observable benefit of a specific local modality like PBT. The heterogeneity in baseline molecular profiles across the included studies introduces “background noise” that statistical adjustments cannot fully eliminate, explaining why clear physical advantages struggle to demonstrate a statistically distinct long-term survival wedge.

### 4.8. Clinical Heterogeneity and Treatment Intent

We acknowledge that pooling patients treated with SBRT (early-stage) and definitive chemoradiotherapy (locally advanced) introduces clinical heterogeneity. Ideally, separate meta-analyses stratified strictly by treatment intent would be methodologically preferable to isolate modality-specific effects without the confounding influence of systemic therapies or varying biological aggressiveness. However, given the limited number of eligible comparative PBT studies currently available, such granular stratification would likely be statistically underpowered. We attempted to address this by performing subgroup analyses based on clinical stage (Stage I vs. Stage I–II), but we recognize that residual differences in systemic therapy use and radiotherapy intent across included studies may still dilute specific outcomes.

## 5. Limitation and Conclusions

Our study has several limitations, including the predominance of retrospective data. A significant limitation is the potential for selection bias inherent in retrospective observational studies. In clinical practice, PBT is often preferentially offered to older patients or those with significant comorbidities who are deemed unfit for photon-based radiotherapy. This ‘negative selection’ could theoretically bias results against PBT. Conversely, socioeconomic factors might select for patients with better access to care. While our pooled analysis suggests a 1-year survival signal despite these potential confounders, residual baseline imbalances cannot be fully excluded.

Furthermore, heterogeneity in toxicity reporting represents another challenge. The lack of standardized endpoints and inconsistent reporting of lower-grade or late toxicities limits our ability to fully characterize the safety profile of PBT compared to photon therapy. Consequently, the true burden of treatment-related morbidity may be underestimated in the current literature.

In conclusion, this meta-analysis bridges the gap between dosimetric theory and clinical reality. While long-term oncologic outcomes appear comparable, exploratory analyses suggest that PBT may be associated with improved 1-year survival. This advantage is likely multifactorial, driven by the sparing of OARs, preservation of nutritional and immune status, and reduction in non-cancer mortality. These findings are hypothesis-generating and support the use of PBT for patients at high risk of toxicity and advocate for a model-based approach to patient selection.

## Figures and Tables

**Table 1:** Characteristics of the included studies.

**Study [Ref.]**	**Country**	**Study Design**	**Data Source**	**Study Period**	**Total Number of Patients**	**Comparison Groups**	**Modality**	**Dose and Fractionation (Total Dose, Dose per Fraction, Total Fractions)**	**Age, Years**	**Male (%)**
Bae et al. [21]	South Korea	Retrospective	Single tertiary institution (Samsung Medical Center)	01/2010–12/2019	202	Photon: 168 Proton: 34	Photon: photon SBRT (3D-CRT, IMRT) Proton: proton SBRT (passive scattering, IMPT)	60 Gy in 4 fractions for all patients	Median 75, IQR 70–79	79.7%
Suh et al. [22]	South Korea	Retrospective	Two institutions (National Cancer Center, Samsung Medical Center)	02/2015–06/2019	289	Photon: 177 Proton: 112	Photon: SABR Proton: PBT	The median total dose was 60 Gy (range 48–70), fraction 4 (range 4–22), and a BED_10_ of 150 CGE (range 78–150).	Median 76, IQR 72–80	79.6%
Yu et al. [28]	United States	Retrospective	Multi-site institution (Mayo Clinic Phoenix, Rochester, NYU Langone Health)	05/2015–08/2018	163	Photon: 128 Proton: 35	Photon: IMRT (volumetric/fixed-beam) Proton: IMPT (pencil-beam active scanning)	The median dose was 60 Gy [RBE] (range 45–72) with a median of 30 fractions (range 15–36).	Mean: 67.0	52.1%
Yu et al. [25]	United States	Retrospective	Single institution (Mayo Clinic, Arizona)	03/2016–06/2018	79	Photon: 46 Proton: 33	Photon: IMRT (volumetric or fixed-beam) Proton: IMPT (pencil-beam active scanning)	The median dose was 60 Gy [RBE] (45–72) with a median of 30 fractions (range 10–39); median daily dose was 2 Gy (range 1.9–5) in the Proton group and 2 Gy (range 1.5–2) in the Photon group	Mean: 72.0	55.7%
Kim et al. [23]	South Korea	Retrospective	Single institution (Samsung Medical Center)	2010/01–2017/10	30	Photon: 22 Proton: 8	Photon: SBRT, 3D-CRT, IMRT Proton: SBPT, IMPT	Photon: 60 Gy in 20 fractions (BED_10_ = 78 Gy) or 15 fractions (BED_10_ = 84 Gy) for X-ray, or 60 Gy in 4 fractions for SBRT Proton: 60–64 CcGE in 4–8 fractions for SBPT, or 60 CcGE in 20 fractions for IMPT	Median 76, range 62–85	96.7%
Nantavithya et al. [29]	United States	RCT	Single institution clinical trial	NA (trial closed early; publication in 2018)	19	Photon: 9 Proton: 10	Photon: SBRT (3D-CRT or IMRT) Proton: SBPT (passive scattering)	50 Gy (RBE) in 4 fractions (12.5 Gy [RBE]/fraction) to PTV in both arms	NA	NA
Higgins et al. [18]	United States	Database-based	National Cancer Database (NCDB)	2004–2012	243,822	Photon: 243,474 Proton: 348	Photon: IMRT, Photons, 3D-CRT, External Beam NOS Proton: PBT	The median dose was 59.4 Gy for the non-proton group, and 60 Gy for the proton group; the median of fractions was 30.	Median 68, range 18–90	56.8%
**Study [Ref.]**	**Clinical Stage**	**Tumor Location**		**Other Baseline Factors (Comorbidities, Lung Function, ECOG Status, If Reported)**				**Concurrent or Adjuvant Therapy (Chemotherapy, Immunotherapy, etc.)**	**Follow-Up Duration, Months**	
Bae et al. [21]	Stage I	Left lower lobe: 16.8% Left upper lobe: 27.2% Right lower lobe: 20.3% Right middle lobe: 3.5% Right upper lobe: 32.2%		ECOG PS 0–1: 82.7%; comorbidities—COPD 42.1% (Photon 36.3%, Proton 70.6%); ILD 12.4% (Photon 10.7%, Proton 20.6%); baseline FEV1: mean 77.85% predicted; baseline DLCO: mean 68.98% predicted; histology—adenocarcinoma 38.1%, squamous cell carcinoma 23.3%, other 6.4%, unproven 32.2%				NA	Median 2.6 years	
Suh et al. [22]	Stage I	Peripheral: 19.4% Close to chest wall: 54.3% Central: 26.3%		ECOG PS 0–1: 85.8%, 2–3: 14.2%; Charlson comorbidity index ≥ 2: 50.9%; chronic lung disease 60.9%; histology—squamous cell carcinoma 30.8%, adenocarcinoma 30.5%, others 3.8%, unproven 34.9%				NA	Median 27, IQR 17.7–39	
Yu et al. [28]	Stage III	NA		ECOG PS 0–1: ~80%; smoking status—majority: former/current smokers; comorbidities—CAD ~20%, COPD ~ 43%, ILD ~ 10%				Concurrent chemotherapy: Photon 87%, Proton 74%; adjuvant immunotherapy: ~31% in both arms	Median 25.5, range 4.6–58.1	
Yu et al. [25]	Stage I–IV (majority: stage III 79%)	NA		ECOG 0–1: ~76%; concurrent chemotherapy 65%; oxygen-dependent COPD: Photon 2%, Proton 18%; reirradiation—Photon 9%, Proton 27%; histology—Photon: adenocarcinoma 70%, squamous cell carcinoma 26%, other 4%; Proton: adenocarcinoma 36%, squamous cell carcinoma 58%, other 6%				Concurrent chemotherapy: Photon 70%, Proton 58%	Median 10.5, range 1–27	
Kim et al. [23]	Stage I–II	NA		ECOG PS 0–1: 21 (70%), 2–3: 9 (30%); GAP index: stage I 10 (33.3%), II–III 20 (66.7%); majority was current or ex-smokers.				NA	Median 11, range 2–51	
Nantavithya et al. [29]	Stage I–II, with high-risk features (centrally located or <5 cm T3 tumor, or isolated lung parenchymal recurrences)	Centrally located, or isolated lung parenchymal recurrence		NA				NA	Median 32	
Higgins et al. [18]	Stage I–IV (majority: stage III 49.8%)	Main bronchus: 6.2% Upper lobe of lung: 57.2% Middle lobe of lung: 3.7% Lower lobe of lung: 22.7% Overlapping lesion of lung: 1.5% Lung, NOS: 8.8%		Charlson–Deyo comorbidity score: 0 (61.7%), 1 (26.9%), ≥2 (11.4%); histology—adenocarcinoma 30.6%, squamous cell carcinoma 37.6%, other 31.8%; 68.4% received chemotherapy; 12.6% underwent surgery.				NA	Photon: median 59.5 Proton: median 39.6	

Abbreviations: RCT, randomized controlled trial; SBRT, stereotactic body radiation therapy; SBPT, stereotactic body proton therapy; SABR, stereotactic ablative radiotherapy; 3D-CRT, three-dimensional conformal radiation therapy; IMRT, intensity-modulated radiation therapy; IMPT, intensity-modulated proton therapy; PBT, proton beam therapy; NOS, not otherwise specified; BED, biologically effective dose; CGE, cobalt gray equivalents; RBE, relative biological effectiveness; PTV, planning target volume; ECOG PS, Eastern Cooperative Oncology Group performance status; COPD, chronic obstructive pulmonary disease; ILD, interstitial lung disease; FEV1, forced expiratory volume in 1 s; DLCO, diffusion capacity of lung for carbon monoxide; CAD, coronary artery disease; NA, not available.

**Table 2:** Radiation pneumonitis and other specific toxicities of the included studies.

Study [Ref.]	Radiation Pneumonitis	Other Specific Toxicities
Bae et al. [21]	Grade ≥ 2: Photon 19.6%, Proton 26.4% Grade ≥ 3: Photon 11.9%, Proton 17.6%	Musculoskeletal grade ≥ 2: Photon 13.7%, Proton 5.9% Skin grade ≥ 2: Photon 4.2%, Proton 0%
Suh et al. [22]	Grade 2—Photon 10.8%, Proton 4.3% Grade 3—Photon 1.1%, Proton 3.2%	Rib fracture—Photon 16.2%, Proton 4.3% Non-cardiac chest pain and chest wall pain—Photon 24.7%, Proton 15.1%
Yu et al. [28]	Grade ≥ 3: Photon: 11.2%; Proton: 0%	NA
Yu et al. [25]	Grade ≥ 3: Photon 2.2%, Proton 6.1%	Grade 3 subacute toxicities (3 months): Esophagitis—Photon 0 patient, Proton 2 patients (*p* = 0.56) Dyspnea—Photon 3 patients, Proton 1 patient (*p* = 0.64)
Kim et al. [23]	Severe treatment-related pulmonary complications—Photon 40.9%, Proton 12.5% (*p* = 0.222)	NA
Nantavithya et al. [29]	NA	One patient in the Proton group developed grade 3 skin fibrosis; no grade 4/5 toxicities reported
Higgins et al. [18]	NA	NA

Abbreviation: NA, not available.

**Table 3:** Sensitivity analyses for overall survival, progression-free survival, and local progression-free survival.

Leave-One-Out	Hazard Ratio	Lower Limit	Upper Limit	Z-Value	*p*-Value
Overall survival					
Bae et al. [21]	0.89	0.64	1.22	−0.73	0.464
Suh et al. [22]	0.85	0.65	1.11	−1.20	0.229
Yu et al. [28]	0.86	0.64	1.14	−1.07	0.286
Yu et al. [25]	0.91	0.66	1.24	−0.62	0.535
Kim et al. [23]	0.94	0.71	1.24	−0.43	0.665
Higgins et al. [18]	1.08	0.79	1.46	0.47	0.637
Pooled estimate	0.91	0.69	1.19	−0.70	0.483
Progression-free survival					
Bae et al. [21]	1.06	0.75	1.51	0.32	0.748
Suh et al. [22]	1.14	0.79	1.65	0.69	0.488
Yu et al. [28]	1.03	0.72	1.47	0.14	0.887
Yu et al. [25]	1.13	0.83	1.55	0.77	0.439
Pooled estimate	1.09	0.81	1.47	0.57	0.572
Local progression-free survival					
Bae et al. [21]	0.69	0.39	1.22	−1.29	0.197
Suh et al. [22]	0.89	0.36	2.16	−0.26	0.791
Yu et al. [28]	0.93	0.35	2.45	−0.15	0.880
Yu et al. [25]	1.06	0.58	1.94	0.18	0.854
Pooled estimate	0.89	0.47	1.69	−0.34	0.732

Results which significantly altered the direction of the effect or the statistical significance after exclusion of a specific study are shown in bold; however, no such changes were observed in this analysis.

## Data Availability

The data that support the findings of this study are available on reasonable request from the corresponding author.
